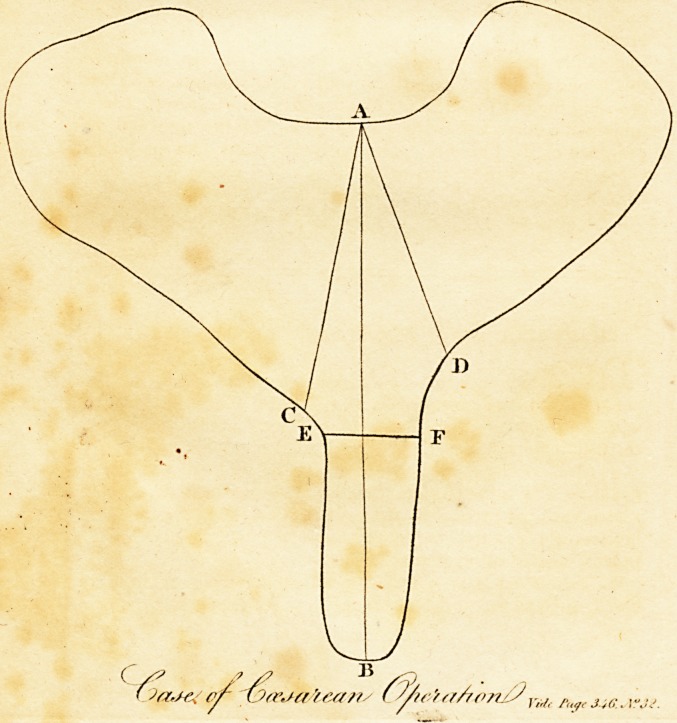# Mr. Wood's Case of Cæsarean Operation

**Published:** 1801-10

**Authors:** W. Wood

**Affiliations:** Manchester


### *To the Editors of the Medical and Phyſical Journal.*

Gentlemen,

If the following Caſe ſhould be thought deſerving a place in your uſeful Journal, be ſo good as to inſert it.

*Manchester*, I am, &c.

*September* 18, 1801. W. WOOD.

---

Hannah Rheubottom, aged forty-one, had been married eighteen years, and borne ſeven children at the full time, in nine years, five of which were living and two dead; one of the latter was afflicted with hydrocephalus, and the crotchet was neceſſary for its extraction; the reſt of her labours were natural and tolerably good, and ſhe had recovered very well in all her lyings-in. She ſuckled five of her children till ſhe was in the third month of pregnancy. Soon after her fourth lying-in ſhe grew lame, and was lame during the time ſhe nurſed three of her laſt children. The lameneſs conſtantly abated, and went off in a few weeks after weaning. She became pregnant of her

---

(1.) Rondelet Aquat. p. 236. Gesner Ibid. 185. Buffon, tom. viii. &c.

(2.) Tyson Philoſ. Tranſact. no. 153. Deſmarchais Voyage, tom. iii. p. 296. Bancroft Guian, p. 125.

her feventh child in the beginning of Auguft, 1794, and was delivered May 12, 1795, of a living child, which fhe fuckled one year and a half, till it died of the fmall-pox. About the ninth month of fuckling fhe began to complain of flying pains in her back, hips, and extremities, which fhe judged to be rheumatic, and became fo lame as fcarcely to be able to walk. She was alfo much troubled with fluor albus. After the death of her child her health improved rapidly, for in one month her health was fully reftored. She enjoyed a good ftate of health afterwards. Her hufband left her for about fix years, and on his return fhe became pregnant for the eighth time in November, 1800; fhe was very healthy during her pregnancy, and able to follow her employment as a fuftian cutter, till within a week of her delivery.

She began to be in labour about 3 P. M. on Wednefday the 12th of Auguft; on Thurfday her pains were lefs forcible, and fhe flept tolerably that night; her pains grew ftrong about noon on Friday, and fhe perceived a difcharge of water in fmall quantity, and unattended with hæmorrhage.

She fent for Mrs. S. the midwife about three in the afternoon, who perceived the deformity of her pelvis, and I was called in about nine in the evening. Finding the deformity fo great as to prevent the introduction of my hand, and the child prefenting with one hand, I immediately called a confultation, and Dr. Hull, Mr. White, and Mr. Thorp, met me at the patient's houfe about eleven the fame evening. We all agreed as to the impoffibility of a delivery per vaginam; and though we could not be certain that the child was alive, as the mother had not felt it move for about two hours before I was called in, and there was no very evident motion of the fingers, we agreed unanimoufly on the propriety and neceffity of the operation of Hyfterotomy; which being propofed, the patient and her friends after fome deliberation agreed to it, and the operation was performed about one o'clock, an anodyne draught being firft given, containing thirty drops of tincture of opium.

Previoufly to the operation, the patient did not appear to be endangered, her pulfe beating only about 86 to 92 ftrokes in a minute; fhe had no vomiting, and fhe had paffed both fæces and urine naturally and regularly. Her pains were at this time ftrong and frequent. There was no tenfion or tendernefs of the abdomen. The tongue was clean.

The incifion was made nearly in a tranfverfe direction on the left fide of the abdomen, about five inches in length, beginning at the umbilicus. This part was fixed upon becaufe the nates of the child could be felt there, and it was evident that no inteftine was interpofed betwixt the abdominal parietes and

 the the uterus. There was ſcarcely any effuſion of blood either from the external wound or from that of the uterus, though the latter was made directly upon the placenta. Inſtead of dividing the placenta, I introduced my hand betwixt it and the uterus, and laying hold of one of the knees of the child, I extracted it with eaſe. My hand paſſed with eaſe betwixt the placenta and uterus; this produced a hæmorrhage, but not in any conſiderable degree, for the whole quantity of blood loſt during the operation did not appear to me to exceed ſeven or eight ounces. After the uterus was emptied, the inteſtines and omentum protruded at the wound; theſe being reduced the integuments were brought into contact, and kept ſo by the interrupted ſuture, the ſtitches of which were made about an inch aſunder, and by ſtraps of adheſive plaſter. Dreſſings and a bandage of flannel being applied, ſhe was placed in bed. She bore the operation very well, ſhe neither fainted nor vomited, and declared that ſhe had ſuffered more pains in a former labour. Her pulſe, which was about 100, immediately after the operation fell to 88, and was moderately ſtrong when we left her at two o'clock. A diſcharge per vaginam immediately took place, and ſhe felt pains on the right ſide of the abdomen ſimilar to after-pains. A pill containing a grain of opium, was ordered to be taken in three hours, provided the pains remained urgent. Saturday, 10 A. M. ſhe had taken the pill, and had got ſome ſleep. Her pulſe 88, moderately ſtrong; tongue clean. The diſcharge per vaginam natural; there had been a moderate diſcharge from the wound. The abdomen not tumefied or tenſe; pains reſembling after-pains felt more on the right ſide of the belly. The pill with opium was ordered to be repeated, and a common clyſter to be given, and repeated in four hours if ſhe ſhould have no evacuation by ſtool.

Seven, P. M. She had made water this evening, but had paſſed no fæces, though two clyſters had been given: Pulſe from 130 to nearly 140, rather ſmall; diſcharge per vaginam free and freſh; the wound had diſcharged a little ſince morning; belly diſtended but not tender; ſkin moiſt, and ſcarcely hotter than natural; no head-ache, or vomiting, or rigors. The clyſters were ordered to be repeated.

Sunday, 16th Aug. 10 A. M. Pulſe 132, ſmall and rather hard; tongue had a ſlight white fur; heat ſcarcely greater than natural to the touch; ſkin moiſt; conſiderable faintneſs and ſickneſs; ſhe had vomited very often without much exertion; had paſſed her urine naturally, and had had two rather liquid ſtools; had experienced no head-ach or rigors; the diſcharge from the vagina natural in appearance and quantity; ſome diſcharge from the wound when ſhe lies on the left ſide. The wound was dreſſed dreſſed this morning, and looked well. Eight leeches and an anodyne fomentation were ordered to be applied to the abdomen. Five grains of calomel and one of purified opium in form of a pill were directed to be given immediately, and a purgative clyſter to be thrown up; and if an evacuation ſhould not be procured in four hours, the clyſter and pill, with half the quantity of opium, were to be repeated. A ſaline julep was likewiſe preſcribed.

Eight, P. M. The vomiting had continued; her pulſe 140, ſmall; tongue clean, ſkin moiſt; not hotter than natural; no rigors; the diſcharge per vaginam of the natural appearance and quantity; the ſtate of the abdomen as in the morning. As ſhe had had no ſtools, the purgative clyſter was ordered to be repeated, and alſo the following pills were directed. Take of calomel eight grains, of purified opium two grains, conſerve a ſufficient quantity; divide into four pills, and take one every four hours.

17th, 9 A. M. She had ſlumbered at times; the pulſe from 132 to 140; ſkin and tongue moiſt; had experienced no rigors or increaſed heat; the diſcharge per vaginam freſh and in ſufficient quantity; the wound had diſcharged but little; on taking off the dreſſings, a portion of inteſtine appeared in contact with the lips of the external wound, but did not protrude betwixt the ligatures; a ſmall portion of omentum alſo appeared at the angle neareſt the umbilicus. The abdomen was tumefied, but was neither very tenſe nor very tender. Her bowels had not been properly evacuated, though ſhe had taken the whole of the pills during the night, and a clyſter had been adminiſtered. Her ſpirits appeared better, and ſhe was leſs faint, but the vomiting was ſtill urgent. She was ordered to take two drachms of Rochelle ſalts, diſſolved in mint water, every hour, till her bowels were opened.

8th, P. M. She had paſſed four looſe and copious ſtools, and became very faint after them; ſhe became delirious; her pulſe was ſo frequent as ſcarcely to be numbered, and very ſmall and weak; her extremities cold and clammy; the abdomen painful, tumefied, and tender; her reſpiration laborious; ſhe had hiccuped, but this ſymptom had not been urgent; the vomiting ſtill continued.

About twelve o'clock ſhe died.

### DISSECTION.

On Tueſday the 18th, about eleven in the forenoon, Dr. Hull and I called upon her huſband, to aſk permiſſion to inſpect the body. We could not obtain leave to open the body, but were permitted to examine the wound. On cutting the ligatures, tures, we found that the lips of the wound did not at all adhere to each other. A portion of ſmall inteſtine, very much diſtended with air, was placed horizontally in the direction of the wound, and in cloſe contact with it; and at the angle near the navel, there was a ſmall portion of omentum. We dragged the ſmall inteſtines out of the wound, and found them very much inflamed; the meſentery was very highly inflamed. The colon, as far as we could trace it, and the omentum, were alſo very much inflamed. There was little, if any, inflammation of that part of the peritoneum which lined the abdomen near the wound.

The uterus lay on the right ſide of the belly, and had deſcended in part into the pelvis; the cervix paſſed through the ſuperior aperture, ſo as to be felt per vaginam. We removed the uterus, and, on examining it carefully at my houſe, we found that the wound was ſituated in the higheſt part of the fundus, and that its lips were livid, and ſhewed a tendency to gangrene. The peritoneal coat of the uterus was rather reddened poſteriorly, but not anteriorly.

The tubæ Fallopinæ, eſpecially the right, were highly inflamed. On cutting open the womb, we found its cervix very much contuſed nearly through the whole ſubſtance, and gangrenous on the inner ſurface. The quantity of coagulated blood in the cavity of the abdomen was very ſmall, it did not appear to equal an ounce.

We examined the pelvis very carefully, both from the abdominal wound and per vaginam, after the removal of the uterus, and eſtimated the dimenſions as follow:

The ſuperior aperture, which was of a triangular form, meaſured from the os ſacrum to the ſymphyſis pubis, ſee the plate, A B, about 3 ½ inches from behind to before; in the direction A C, about two inches; and leſs in the remainder of the ſpace on the right ſide; from behind to before, in the direction A D, about one inch and a half, and ſtill leſs in the remaining part on the left ſide; from the part where the oſſa pubis were bent and approximated, E F, the ſpace from one os pubis to the other, quite to the ſymphyſis, meaſured rather more than half an inch. The inferior aperture was much more contracted in proportion than the ſuperior. Immediately under the ſymphyſis pubis, the ſpace from one ramus to the other meaſured about an inch; lower down, the ſpace from one ramus iſchii to the other ſcarcely meaſured half an inch. From the tuberoſity of one os iſchium to the other, at their moſt diſtant points, the ſpace was about 1 ½ inch. From the tuberoſity of one os iſchium to the apex of the os coccygis, it meaſured about 1 ½ inch on each ſide.

From

From the apex of the os coccygis to the approximation of the rami iſchii, nearly two inches; from the apex of the os ſacrum to the point where the rami iſchii approximated ſo much as to barely admit one finger, nearly three inches.

The os ſacrum was very much bent; the oſſa pubis had each a ſharp prominent ridge, about an inch in length, on their margins, at the ſuperior aperture.

The vertebræ were bent forwards, ſo as to render the back very hollow; but there was no other diſtortion of the ſpine: The clavicles were very much bent, inſomuch, that ſhe once applied to a bone ſetter, thinking they had been broken; the ribs appeared flattened; the ſternum projected more than is uſual, and was rather incurvated. None of the other bones were altered in their form.

### Observations.

The deformity above alluded to, was produced in the adult ſtate by *malacosteon*; and it is very extraordinary, that five caſes of this diſeaſe have occurred to me in the ſpace of fourteen months The firſt was that of Eliz. Thompſon, upon whom I performed the Cæſarean operation: Her caſe is given by me in the *Appendix to the fifth vol. of the Memoirs of the Medical Society.* The ſecond and third caſes were thoſe of Hannah Davies of Bradſhaw Street, and Jane Kinnerly of Water Street, Mancheſter; the former was delived by the crotchet, and the latter died undelivered; their caſes are mentioned by Dr. Hull, in the Appendix to his Tranſlation of the *Memoirs on the Cæsarean Operation, by M. Baudelocque.* The fourth was that of Mary Robinſon, of Deans-gate, Mancheſter; ſhe has had ſeven children born at the full time. I attended her in her two laſt labours; the former happened about three years ago; at that time there was no diſtortion of the pelvis. She began to be in labour the latter time, on the of May; I was ſent for in the evening, ſhe appeared much emaciated, and informed me, that ſhe had been in a poor ſtate of health for ſix months, during which time ſhe had been affected with pains in her back and hips, which ſhe judged to be rheumatic, and was become ſo lame, as ſcarcely to be able to walk acroſs the room: Her legs and thighs were very much ſwelled, and quite anaſarcous: Her pains had been frequent moſt of the day. Upon examination, per vaginam, I found the os uteri fully dilated, and the head of the fœtus preſenting. No part of the cranium was then forced into the ſuperior aperture of the pelvis. I found the pelvis ſo much deformed, that it was neceſſary to open the head, and deliver by the crotchet, which was accompliſhed with ſome difficulty. The ſuperior aperture of the pelvis, in its wideſt wideſt ſpace, appeared to me to be little more than two inches: the patient recovered tolerably well, and is now able to walk about, and enjoys a pretty good ſtate of health. The fifth caſe was that of Hannah Rheubottom, in which it appears, that the affection of the bones took place during the time ſhe was a nurſe, and when ſhe left off ſuckling, the progreſs of the diſeaſe was ſtopped. It ſeems remarkable, that the contuſion the cervix uteri had undergone, by the effects of the labour pains, ſhould not have produced ſome alarming ſymptoms previous to the delivery.

## Figures and Tables

**Figure f1:**